# Supplementary material for: Integrative machine learning analysis of multiple gene expression profiles in cervical cancer
Source: PeerJ. 2018 Jul 25;6:e5285. doi: 10.7717/peerj.5285 (PMC6064203; doi:10.7717/peerj.5285)
Supplement: Appendix S3 [file peerj-06-5285-s003.docx]

Result of the Annotation of the 9 upregulated genes with GSEA.

|  | **Gene Set** | **Gene** | **ES** | **NES** | **NOM p-val** | **FDR q-val** | **Description** |
| --- | --- | --- | --- | --- | --- | --- | --- |
|  | [MODULE_455](http://www.broadinstitute.org/gsea/msigdb/cards/MODULE_455) | SPON1 | 1.00 | 1.60 | 0.000 | 0.000 | cancer module 455. |
|  | [MORF_RAN](http://www.broadinstitute.org/gsea/msigdb/cards/MORF_RAN) | RPLP2 | 0.88 | 1.40 | 0.000 | 0.000 | Neighborhood of RAN, member RAS oncogene family in the MORF expression compendium. |
|  | [MODULE_147](http://www.broadinstitute.org/gsea/msigdb/cards/MODULE_147) | RPLP2 | 0.88 | 1.40 | 0.000 | 0.000 | G-Protein-coupled (GCPR) family. |
|  | [MODULE_195](http://www.broadinstitute.org/gsea/msigdb/cards/MODULE_195) | RPLP2 | 0.88 | 1.40 | 0.000 | 0.000 | Breast cancer expression clusters. |
|  | [MODULE_1](http://www.broadinstitute.org/gsea/msigdb/cards/MODULE_1) | SPON1, PEG3 | 0.86 | 1.33 | 0.000 | 0.053 | Ovary genes. |
|  | [MODULE_47](http://www.broadinstitute.org/gsea/msigdb/cards/MODULE_47) | SPON1 | 1.00 | 1.33 | 0.000 | 0.044 | ECM and collagens. |
|  | [MODULE_342](http://www.broadinstitute.org/gsea/msigdb/cards/MODULE_342) | SPON1 | 1.00 | 1.28 | 0.000 | 0.107 | cancer module 342 |
|  | [MORF_EIF3S6](http://www.broadinstitute.org/gsea/msigdb/cards/MORF_EIF3S6) | RPLP2 | 0.88 | 1.27 | 0.000 | 0.094 | Neighborhood of EIF3S6 eukaryotic translation initiation factor 3, subunit 6 48kDa in the MORF expression compendium. |
|  | [MODULE_114](http://www.broadinstitute.org/gsea/msigdb/cards/MODULE_114) | RPLP2 | 0.88 | 1.27 | 0.000 | 0.083 | Protein biosynthesis and ribosomes. |
|  | [MORF_NME2](http://www.broadinstitute.org/gsea/msigdb/cards/MORF_NME2) | RPLP2 | 0.88 | 1.22 | 0.000 | 0.244 | Neighborhood of NME2 non-metastatic cells 2, protein (NM23B) expressed in in the MORF expression compendium. |
|  | [MORF_BECN1](http://www.broadinstitute.org/gsea/msigdb/cards/MORF_BECN1) | RPLP2 | 0.88 | 1.17 | 0.000 | 0.496 | Neighborhood of BECN1 beclin 1 (coiled-coil, myosin-like BCL2 interacting protein) in the MORF expression compendium. |
|  | [MORF_ACP1](http://www.broadinstitute.org/gsea/msigdb/cards/MORF_ACP1) | RPLP2 | 0.88 | 1.17 | 0.000 | 0.455 | Neighborhood of ACP1 acid phosphatase 1, soluble in the MORF expression compendium. |
|  | [MORF_UBE2I](http://www.broadinstitute.org/gsea/msigdb/cards/MORF_UBE2I) | RPLP2 | 0.88 | 1.17 | 0.000 | 0.420 | Neighborhood of UBE2I ubiquitin-conjugating enzyme E2I (UBC9 homolog, yeast) in the MORF expression compendium. |
|  | [MODULE_83](http://www.broadinstitute.org/gsea/msigdb/cards/MODULE_83) | RPLP2 | 0.88 | 1.17 | 0.000 | 0.390 | cancer module 83. |
|  | [MODULE_150](http://www.broadinstitute.org/gsea/msigdb/cards/MODULE_150) | RPLP2 | 0.88 | 1.17 | 0.000 | 0.364 | translation elongation. |
|  | [MODULE_491](http://www.broadinstitute.org/gsea/msigdb/cards/MODULE_491) | RPLP2 | 0.88 | 1.17 | 0.000 | 0.341 | cancer module 491 |
|  | [MODULE_418](http://www.broadinstitute.org/gsea/msigdb/cards/MODULE_418) | SPON1 | 1.00 | 1.14 | 0.000 | 0.492 | cancer module 418 |
|  | MORF_TNFRSF25 | BTD | 0.75 | 1.50 | 0.00 | 0.026 | Neighborhood of TNFRSF25 tumor necrosis factor receptor superfamily, member 25 in the MORF expression compendium. |
|  | MORF_FOSL1 | BTD | 0.75 | 1.20 | 0.00 | 0.693 | Neighborhood of FOSL1 FOS-like antigen 1 in the MORF expression compendium |
|  | [MORF_CDC2L5](http://www.broadinstitute.org/gsea/msigdb/cards/MORF_CDC2L5) | BTD | 0.75 | 1.13 | 0.333 | 0.891 | Neighborhood of CDC2L5 cell division cycle 2-like 5 (cholinesterase-related cell division controller) in the MORF expression compendium. |
|  | [MODULE_19](http://www.broadinstitute.org/gsea/msigdb/cards/MODULE_19) | PEG3 | 0.75 | 1.13 | 0.000 | 0.479 | Adrenal gland - metabolic genes. |
|  | [MORF_ACTG1](http://www.broadinstitute.org/gsea/msigdb/cards/MORF_ACTG1) | RPLP2 | 0.88 | 1.11 | 0.000 | 0.553 | Neighborhood of ACTG1 actin, gamma 1 in the MORF expression compendium. |
|  | [GCM_TPT1](http://www.broadinstitute.org/gsea/msigdb/cards/GCM_TPT1) | RPLP2 | 0.88 | 1.11 | 0.000 | 0.525 | Neighborhood of TPT1 tumor protein, translationally-controlled 1 in the GCM expression compendium. |
|  | [MORF_MT4](http://www.broadinstitute.org/gsea/msigdb/cards/MORF_MT4) | BTD | 0.75 | 1.06 | 0.333 | 1.000 | Neighborhood of MT4 metallothionein IV in the MORF expression compendium. |
|  | [MORF_HEAB](http://www.broadinstitute.org/gsea/msigdb/cards/MORF_HEAB) | BTD | 0.75 | 1.04 | 0.500 | 1.000 | Neighborhood of HEAB - in the MORF expression compendium. |
|  | [MORF_RBBP8](http://www.broadinstitute.org/gsea/msigdb/cards/MORF_RBBP8) | BTD | 0.75 | 1.03 | 0.400 | 0.948 | Neighborhood of RBBP8 retinoblastoma binding protein 8 in the MORF expression compendium |
|  | [MORF_ATRX](http://www.broadinstitute.org/gsea/msigdb/cards/MORF_ATRX) | BTD | 0.75 | 1.00 | 0.500 | 1.000 | Neighborhood of ATRX alpha thalassemia/mental retardation syndrome X-linked (RAD54 homolog, S. cerevisiae) in the MORF expression compendium. |
|  | [MORF_ERCC2](http://www.broadinstitute.org/gsea/msigdb/cards/MORF_ERCC2) | BTD | 0.75 | 1.00 | 0.333 | 1.000 | Neighborhood of ERCC2 excision repair cross-complementing rodent repair deficiency, complementation group 2 (xeroderma pigmentosum D) in the MORF expression compendium. |
|  | [MORF_FDXR](http://www.broadinstitute.org/gsea/msigdb/cards/MORF_FDXR) | BTD | 0.75 | 1.00 | 0.000 | 1.000 | Neighborhood of FDXR ferredoxin reductase in the MORF expression compendium. |
|  | [MORF_DDX11](http://www.broadinstitute.org/gsea/msigdb/cards/MORF_DDX11) | BTD | 0.75 | 1.00 | 0.333 | 0.941 | Neighborhood of DDX11 DEAD/H (Asp-Glu-Ala-Asp/His) box polypeptide 11 (CHL1-like helicase homolog, S. cerevisiae) in the MORF expression compendium. |
|  | [MORF_PPP5C](http://www.broadinstitute.org/gsea/msigdb/cards/MORF_PPP5C) | BTD | 0.75 | 1.00 | 0.500 | 0.878 | Neighborhood of PPP5C protein phosphatase 5, catalytic subunit in the MORF expression compendium. |
|  | [MORF_FANCG](http://www.broadinstitute.org/gsea/msigdb/cards/MORF_FANCG) | BTD | 0.75 | 0.96 | 0.250 | 0.795 | Neighborhood of FANCG Fanconi anemia, complementation group G in the MORF expression compendium. |
|  | [MORF_PML](http://www.broadinstitute.org/gsea/msigdb/cards/MORF_PML) | BTD | 0.75 | 0.90 | 0.667 | 0.825 | Neighborhood of PML promyelocytic leukemia in the MORF expression compendium. |
|  | MORF_TPR | BTD | 0.75 | 0.89 | 0.750 | 0.793 | Neighborhood of TPR translocated promoter region (to activated MET oncogene) in the MORF expression compendium. |
|  | MODULE_247 | BTD | 0.75 | 0.86 | 0.667 | 0.817 | Xenobiotic metabolism. |
|  | MORF_PSMF1 | BTD | 0.75 | 0.75 | 1.000 | 0.882 | Neighborhood of PSMF1 proteasome (prosome, macropain) inhibitor subunit 1 (PI31) in the MORF expression compendium. |
|  | MORF_JAG1 | BTD | 0.75 | NA | NA | 1.00 | Neighborhood of JAG1 jagged 1 (Alagille syndrome) in the MORF expression compendium. |
|  | MODULE_69 | CNKR2 | -0.63 | -0.77 | 0.500 | 0.904 | Genes in the cancer module 69. |
|  | MODULE_136 | CNKR2 | -0.63 | -0.71 | 1.000 | 0.859 | Genes in the cancer module 136. |
|  | GNF2_CCNA1 | SPATA6 | -0.50 | -1.00 | 0.000 | 0.823 | Neighborhood of CCNA1 cyclin A1 in the GNF2 expression compendium |
|  | MODULE_32 | NA | 0.88 | 1.11 | 0.000 | 0.500 | - |
|  | MODULE_55 | NA | 0.75 | 1.09 | 0.000 | 0.528 | - |
|  | MODULE_72 | NA | 0.75 | 1.09 | 0.000 | 0.505 | - |
|  | GNF2_EIF3S6 | NA | 0.88 | 1.08 | 0.250 | 0.511 | - |
|  | GNF2_GLTSCR2 | NA | 0.88 | 1.08 | 0.000 | 0.491 | - |
|  | MODULE_356 | NA | 0.88 | 1.08 | 0.500 | 0.472 | - |
|  | MORF_ANP32B | NA | 0.88 | 1.00 | 0.333 | 1.000 | - |
|  | MORF_ARAF1 | NA | 0.88 | 1.00 | 0.333 | 0.994 | - |
|  | MORF_EIF4A2 | NA | 0.88 | 1.00 | 0.000 | 0.959 | - |
|  | MORF_JUND | NA | 0.88 | 1.00 | 0.500 | 0.927 | - |
|  | MODULE_503 | NA | 0.88 | 1.00 | 0.000 | 0.897 | - |
|  | MODULE_576 | NA | 0.88 | 1.00 | 0.000 | 0.869 | - |
|  | MODULE_568 | NA | 0.88 | 0.97 | 0.400 | 0.869 | - |
|  | GCM_MAP1B | NA | 0.75 | 0.95 | 0.333 | 0.886 | - |
|  | MODULE_12 | NA | 0.75 | 0.92 | 0.500 | 0.918 | - |
|  | GCM_NCAM1 | NA | 0.75 | 0.90 | 0.333 | 0.975 | - |
|  | MODULE_24 | NA | 0.75 | 0.90 | 0.667 | 0.949 | - |
|  | MODULE_212 | NA | 0.64 | 0.88 | 0.667 | 0.948 | - |
|  | MORF_NPM1 | NA | 0.88 | 0.88 | 1.000 | 0.938 | - |
|  | MORF_SART1 | NA | 0.88 | 0.88 | 1.000 | 0.915 | - |
|  | MORF_TPT1 | NA | 0.88 | 0.88 | 1.000 | 0.892 | - |
|  | MODULE_484 | NA | 0.88 | 0.88 | 1.000 | 0.871 | - |
|  | MODULE_88 | NA | 0.75 | 0.80 | 1.000 | 0.972 | - |
|  | GCM_PTPRD | NA | 0.75 | 0.75 | 1.000 | 0.991 | - |
|  | GCM_PTK2 | NA | 0.43 | 0.50 | 1.000 | 1.000 | - |
|  | MORF_CCNI | NA | 0.88 | NA | NA | 1.000 | - |
|  | MODULE_126 | NA | 0.63 | NA | NA | 1.000 | - |
|  | MODULE_429 | NA | 0.88 | NA | NA | 1.000 | - |
|  | MODULE_179 | NA | -0.63 | -0.65 | 1.000 | 0.894 | - |
|  | GCM_MAPK10 | NA | -0.63 | -0.63 | 1.000 | 0.875 | - |
|  | GNF2_MLF1 | NA | -0.50 | -0.60 | 1.000 | 0.846 | - |

Result of the Annotation of the 12 downregulated genes with GSEA.

|  | **Gene Set** | **Gene** | **ES** | **NES** | **NOM p-val** | **FDR q-val** | **Gene Set Description** |
| --- | --- | --- | --- | --- | --- | --- | --- |
|  | [GCM_ZNF198](http://www.broadinstitute.org/gsea/msigdb/cards/GCM_ZNF198) | SLC5A3 | 0.90 | 1.50 | 0.000 | 0.000 | Neighborhood of ZNF198 NULL in the GCM expression compendium. |
|  | [GNF2_CCNA2](http://www.broadinstitute.org/gsea/msigdb/cards/GNF2_CCNA2) | ASF1B | 1.00 | 1.43 | 0.000 | 0.000 | Neighborhood of CCNA2 cyclin A2 in the GNF2 expression compendium. |
|  | [MODULE_54](http://www.broadinstitute.org/gsea/msigdb/cards/MODULE_54) | ASF1B | 1.00 | 1.43 | 0.000 | 0.000 | Cell cycle (expression cluster). |
|  | [GNF2_BUB1](http://www.broadinstitute.org/gsea/msigdb/cards/GNF2_BUB1) | ASF1B | 1.00 | 1.43 | 0.000 | 0.000 | Neighborhood of BUB1 budding uninhibited by benzimidazoles 1 homolog (yeast) in the GNF2 expression compendium. |
|  | [MORF_RAD23B](http://www.broadinstitute.org/gsea/msigdb/cards/MORF_RAD23B) | COPB2, PRDX3, LSM3 | 0.63 | 1.41 | 0.000 | 0.020 | Neighborhood of RAD23B RAD23 homolog B (S. cerevisiae) in the MORF expression compendium. |
|  | [GNF2_MCM4](http://www.broadinstitute.org/gsea/msigdb/cards/GNF2_MCM4) | ASF1B | 1.00 | 1.33 | 0.000 | 0.035 | Neighborhood of MCM4 minichromosome maintenance deficient 4 (S. cerevisiae) in the GNF2 expression compendium. |
|  | [MODULE_563](http://www.broadinstitute.org/gsea/msigdb/cards/MODULE_563) | COPB2 | 0.80 | 1.33 | 0.000 | 0.030 | Genes in the cancer module 563. |
|  | [GNF2_CKS1B](http://www.broadinstitute.org/gsea/msigdb/cards/GNF2_CKS1B) | ASF1B | 1.00 | 1.25 | 0.000 | 0.086 | Neighborhood of CKS1B CDC28 protein kinase regulatory subunit 1B in the GNF2 expression compendium. |
|  | [MODULE_397](http://www.broadinstitute.org/gsea/msigdb/cards/MODULE_397) | ASF1B | 1.00 | 1.25 | 0.000 | 0.077 | cancer module 397. |
|  | [MODULE_99](http://www.broadinstitute.org/gsea/msigdb/cards/MODULE_99) | SLC5A | 0.90 | 1.23 | 0.333 | 0.093 | cancer module 99. |
|  | [MORF_DAP](http://www.broadinstitute.org/gsea/msigdb/cards/MORF_DAP) | PRDX3, LSM3 | 0.56 | 1.16 | 0.333 | 0.184 | Neighborhood of DAP death-associated protein in the MORF expression compendium. |
|  | [GNF2_ESPL1](http://www.broadinstitute.org/gsea/msigdb/cards/GNF2_ESPL1) | ASF1B | 1.00 | 1.15 | 0.000 | 0.187 | Neighborhood of ESPL1 extra spindle poles like 1 (S. cerevisiae) in the GNF2 expression compendium. |
|  | [MODULE_117](http://www.broadinstitute.org/gsea/msigdb/cards/MODULE_117) | SLC5A | 0.90 | 1.13 | 0.000 | 0.284 | Signaling. |
|  | [GNF2_BUB1B](http://www.broadinstitute.org/gsea/msigdb/cards/GNF2_BUB1B) | ASF1B | 1.00 | 1.11 | 0.000 | 0.312 | Neighborhood of BUB1B BUB1 budding uninhibited by benzimidazoles 1 homolog beta (yeast) in the GNF2 expression compendium. |
|  | [MORF_RAC1](http://www.broadinstitute.org/gsea/msigdb/cards/MORF_RAC1) | COPB2 | 0.80 | 1.07 | 0.000 | 0.363 | Neighborhood of RAC1 ras-related C3 botulinum toxin substrate 1 (rho family, small GTP binding protein Rac1) in the MORF expression compendium. |
|  | [MORF_PSMC2](http://www.broadinstitute.org/gsea/msigdb/cards/MORF_PSMC2) | COPB2 | 0.80 | 1.04 | 0.333 | 0.442 | Neighborhood of PSMC2 proteasome (prosome, macropain) 26S subunit, ATPase, 2 in the MORF expression compendium. |
|  | [MORF_RAB5A](http://www.broadinstitute.org/gsea/msigdb/cards/MORF_RAB5A) | COPB2 | 0.80 | 0.94 | 0.500 | 0.695 | Neighborhood of RAB5A, member RAS oncogene family in the MORF expression compendium. |
|  | [MORF_RAB1A](http://www.broadinstitute.org/gsea/msigdb/cards/MORF_RAB1A) | COPB2 | 0.80 | 0.89 | 1.000 | 0.790 | Neighborhood of RAB1A, member RAS oncogene family in the MORF expression compendium. |
|  | GCM_DLG1 | HIGD1A | -1.00 | 1.25 | 0.602 | 1.000 | Neighborhood of DLG1 discs, large homolog 1 (Drosophila) in the GCM expression compendium. |
|  | GNF2_HAT1 | PSMD6 | -0.90 | -1.44 | 0.250 | 0.560 | Neighborhood of HAT1 histone acetyltransferase 1 in the GNF2 expression compendium. |
|  | MODULE_114 | LSM3, RPL15, PSMD6 | -0.64 | -1.39 | 0.000 | 0.565 | Protein biosynthesis and ribosomes. |
|  | MORF_PAPSS1 | PDHB | -0.80 | -1.33 | 0.000 | 0.839 | Neighborhood of PAPSS1 3'-phosphoadenosine 5'-phosphosulfate synthase 1 in the MORF expression compendium. |
|  | GCM_ACTG1 | PSMD6 | -0.90 | -1.29 | 0.000 | 0.715 | Neighborhood of ACTG1 actin, gamma 1 in the GCM expression compendium. |
|  | GCM_BECN1 | PSMD6 | -0.90 | -1.29 | 0.000 | 0.721 | Neighborhood of BECN1 beclin 1 (coiled-coil, myosin-like BCL2 interacting protein) in the GCM expression compendium. |
|  | MORF_HDAC1 | PDHB | -1.25 | 0.200 | 0.696 | 1.000 | Neighborhood of HDAC1 histone deacetylase 1 in the MORF expression compendium. |
|  | MODULE_83 | RPL15, PSMD6 | -0.78 | -1.21 | 0.000 | 0.779 | cancer module 83. |
|  | [MODULE_28](http://www.broadinstitute.org/gsea/msigdb/cards/MODULE_28) | PSMD6 | -0.90 | -1.20 | 0.500 | 0.748 | cancer module 28. |
|  | [MODULE_331](http://www.broadinstitute.org/gsea/msigdb/cards/MODULE_331) | PSMD6 | -0.90 | -1.20 | 0.000 | 0.673 | cancer module 331. |
|  | [MORF_RFC4](http://www.broadinstitute.org/gsea/msigdb/cards/MORF_RFC4) | PDHB | -0.80 | -1.14 | 0.000 | 1.000 | Neighborhood of RFC4 replication factor C (activator 1) 4, 37kDa in the MORF expression compendium. |
|  | [MORF_GNB1](http://www.broadinstitute.org/gsea/msigdb/cards/MORF_GNB1) | PDHB | -0.80 | -1.14 | 0.000 | 0.970 | Neighborhood of GNB1 guanine nucleotide binding protein (G protein), beta polypeptide 1 in the MORF expression compendium. |
|  | [MODULE_91](http://www.broadinstitute.org/gsea/msigdb/cards/MODULE_91) | PSMD6 | -0.90 | -1.13 | 0.000 | 0.952 | Proteasome. |
|  | [MORF_NME2](http://www.broadinstitute.org/gsea/msigdb/cards/MORF_NME2) | RPL15 | -0.70 | -1.11 | 0.000 | 1.000 | Neighborhood of NME2 non-metastatic cells 2, protein (NM23B) expressed in in the MORF expression compendium. |
|  | [MODULE_32](http://www.broadinstitute.org/gsea/msigdb/cards/MODULE_32) | RPL15 | -0.70 | -1.09 | 0.200 | 1.000 | cancer module 32. |
|  | [GCM_DDX5](http://www.broadinstitute.org/gsea/msigdb/cards/GCM_DDX5) | PSMD6 | -0.90 | -1.09 | 0.250 | 0.978 | Neighborhood of DDX5 DEAD (Asp-Glu-Ala-Asp) box polypeptide 5 in the GCM expression compendium. |
|  | [MODULE_363](http://www.broadinstitute.org/gsea/msigdb/cards/MODULE_363) | RPL15 | -0.70 | -1.08 | 0.500 | 0.980 | Genes in the cancer module 363. |
|  | [MORF_PRKDC](http://www.broadinstitute.org/gsea/msigdb/cards/MORF_PRKDC) | PDHB | -0.80 | -1.07 | 0.000 | 1.000 | Neighborhood of PRKDC protein kinase, DNA-activated, catalytic polypeptide in the MORF expression compendium |
|  | [MORF_ACTG1](http://www.broadinstitute.org/gsea/msigdb/cards/MORF_ACTG1) | RPL15 | -0.70 | -1.03 | 0.200 | 1.000 | Neighborhood of ACTG1 actin, gamma 1 in the MORF expression compendium. |
|  | [MODULE_151](http://www.broadinstitute.org/gsea/msigdb/cards/MODULE_151) | LSM3, RPL15, PSMD6 | -0.64 | -1.02 | 0.333 | 1.000 | cancer module 151. |
|  | MORF_AP3D1 | NA | -0.80 | -1.00 | 0.667 | 1.000 | - |
|  | GNF2_FBL | NA | -0.70 | -1.00 | 0.500 | 1.000 | - |
|  | MORF_RAD23A | NA | -0.56 | -0.98 | 0.333 | 1.000 | - |
|  | MORF_EI24 | NA | -0.56 | -0.97 | 0.500 | 1.000 | - |
|  | MORF_XRCC5 | NA | -0.80 | -0.91 | 0.750 | 1.000 | - |
|  | MORF_CSNK2B | NA | -0.50 | -0.90 | 0.667 | 1.000 | - |
|  | GNF2_UBE2I | NA | -0.70 | -0.90 | 0.750 | 1.000 | - |
|  | MORF_PRDX3 | NA | -0.56 | -0.89 | 0.333 | 1.000 | - |
|  | MORF_NPM1 | NA | -0.70 | -0.88 | 1.000 | 1.000 | - |
|  | MORF_TPT1 | NA | -0.70 | -0.88 | 0.667 | 1.000 | - |
|  | GNF2_ST13 | NA | -0.70 | -0.88 | 1.000 | 1.000 | - |
|  | MORF_EIF3S2 | NA | -0.80 | -0.86 | 0.667 | 1.000 | - |
|  | MORF_DAP3 | NA | -0.80 | -0.83 | 1.000 | 1.000 | - |
|  | MORF_SOD1 | NA | -0.50 | -0.80 | 0.667 | 1.000 | - |
|  | MORF_HDAC2 | NA | -0.56 | -0.79 | 1.000 | 1.000 | - |
|  | MODULE_15 | NA | -0.56 | -0.78 | 1.000 | 1.000 | - |
|  | GNF2_EIF3S6 | NA | -0.70 | -0.78 | 1.000 | 1.000 | - |
|  | MORF_BUB3 | NA | -0.56 | -0.77 | 1.000 | 1.000 | - |
|  | MORF_RAD21 | NA | -0.50 | -0.77 | 0.500 | 1.000 | - |
|  | MORF_MAP2K2 | NA | -0.56 | -0.68 | 1.000 | 1.000 | - |
|  | MORF_PPP6C | NA | -0.50 | -0.67 | 1.000 | 1.000 | - |
|  | MORF_SKP1A | NA | -0.50 | -0.67 | 1.000 | 1.000 | - |
|  | MODULE_72 | NA | -0.50 | -0.67 | 0.750 | 1.000 | - |
|  | MORF_PSMC1 | NA | -0.50 | -0.65 | 0.667 | 1.000 | - |
|  | MODULE_8 | NA | -0.50 | -0.65 | 1.000 | 1.000 | - |
|  | MODULE_45 | NA | -0.60 | -0.64 | 1.000 | 1.000 | - |
|  | MORF_HAT1 | NA | -0.50 | -0.63 | 1.000 | 1.000 | - |
|  | MODULE_16 | NA | -0.50 | -0.63 | 1.000 | 1.000 | - |
|  | MODULE_17 | NA | -0.50 | -0.56 | 1.000 | 1.000 | - |
|  | MORF_PPP1CA | NA | -0.50 | -0.53 | 1.000 | 1.000 | - |
|  | MODULE_3 | NA | -0.50 | -0.50 | 1.000 | 0.980 | - |
|  | [MODULE_28](http://www.broadinstitute.org/gsea/msigdb/cards/MODULE_28) | NA | -0.90 | -1.20 | 0.500 | 0.748 | - |
|  | [MODULE_331](http://www.broadinstitute.org/gsea/msigdb/cards/MODULE_331) | NA | -0.90 | -1.20 | 0.000 | 0.673 | - |

Table Description:

1. Gene set: name of the gene sets
2. Gene: genes of interest that matched with the gene set
3. ES: Enrichment Score
4. NES: Normalized Enrichment Score
5. NOM p-value: Nominal p-value
6. FDR q-value: False Discovery Rate value
